# Supplementary material for: The bacterial multidrug resistance regulator BmrR distorts promoter DNA to activate transcription
Source: Nat Commun. 2020 Dec 8;11:6284. doi: 10.1038/s41467-020-20134-y (PMC7722741; doi:10.1038/s41467-020-20134-y)
Supplement: Supplementary file 1 — Supplementary Information [file 41467_2020_20134_MOESM1_ESM.pdf]

Supplementary Tables

Supplementary Table 1. The statistics of the cryo-EM structure of *B. subtilis* BmrR-TAC.

	***B. subtilis* BmrR-TAC** (EMDB-30390) (PDB 7CKQ)
**Data collection and processing**	
Magnification	22,500
Voltage (kV)	300
Electron exposure ($e^{-}/Å^2$)	60.8
Defocus range (μm)	-2.2 to -1.2
Pixel size (Å)	1.0
Symmetry imposed	C1
Initial particle images (no.)	494,295
Final particle images (no.)	103,226
Map resolution (Å)	4.4
FSC threshold	0.143
Map resolution range (Å)	4.0-7.0
**Refinement**	
Initial model used (PDB code)	6LDI, 3Q3D, 4NJC
Model resolution (Å)	4.4
FSC threshold	0.143
Model resolution range (Å)	4.0-7.0
Map sharpening *B* factor ($Å^2$)	-141.5
Model composition	
Non-hydrogen atoms	27,470
Protein residues	3,586
Ligands	5
B factors ($Å^2$)	
Protein	96.04
Ligand	158.30
R.m.s. deviations	
Bond lengths (Å)	0.004
Bond angles (°)	0.779
Validation	
MolProbity score	1.91
Clashscore	9.00
Poor rotamers (%)	0.67
Ramachandran plot	
Favored (%)	93.50
Allowed (%)	6.50
Disallowed (%)	0.00

Supplementary Table 2. The sequences of reported MerR-TF regulated promoters with the -35 and -10 elements underlined.

Name	Promoter sequences	Spacer (bp)	Ref.
Tn501/Tn21 P*mer* (MerR)	TTGACTCCGTACATGAGTACGGAAGTAAGGT	19	[1]
B. subtilis P*mer* (MerR)	TTTACCCTGTACTAAGGTACGTGGTTTATGCT	20	[2]
S. aureus P*mer* (MerR)	TTGACCGTGTACTATGGTACAGGGTTTATACT	20	[3]
E. coli P*zntA* (ZntR)	TTGACTCTGGAGTCGACTCCAGAGTGTATCCT	20	[4]
E. coli P*copA* (CueR)	TTGACCTTCCCCTTGCTGGAAGGTTTAACCT	19	[5]
E. coli P*cueO* (CueR)	TTGACCTTCCCGTAAGGGGAAGGACTATGCT	19	[5]
Synechocystis P*coaT* (CoaR)	TTGACATTGACACTAATGTTAAGGTTTAGGCT	20	[6]
R. metallidurans P*pbrA* (PbrR)	TTGACTCTATAGTAACTAGAGGGTGTTAAAT	19	[7]
P. putida P*cadA* (CadR)	TTGACCCTATAGTGGCTACAGGGTGTTCACTT	20	[8]
E. coli P*soxS* (SoxR)	TTTACCTCAAGTTAACTTGAGGAATTATACT	19	[9]
S.lividans P*tipA* (TipA_L)	TTGCACCTCACGTCACGTGAGGAGGCAGCGT	19	[10]
B. subtilis P*bmr* (BmrR)	TTGACTCTCCCCTAGGAGGAGGTCTTACAGT	19	[11]
B. subtilis P*blt* (BltR)	TTGACTATACGGTAACCATATACCTTATGAT	19	[12]
B. subtilis P*mta* (Mta)	TTGACCCTAACGTTGCGTGATTGTTTACGAT	19	[13]
P. aeruginosa P*brlR* (BrlR)	TTGACCTTGCCCCAGGGGCAATCCGTAGTCT	19	[14]
N. gonorrhoeae P*nmlR* (NmlR)	TTGCCTTAGAGTGAACTCTAAAATGTAAACT	19	[15]
R. sphaeroides P*copA* (CueR)	TTGACCTTCCAGTTGTGGGAAGCCCCATCTT	19	[16]
H. influenzae P*nik* (NimR)	TTGATTCTAAAGTTACTTCATATTTTATCAT	19	[17]
B. subtilis P*adhR* (AdhR)	TTGACTTAAAGTTAACTTTAAGTGTTACCTT	19	[18]
B. pertussis P*bor* (ZccR)	TTGACCCTATAGTAACTCCAGGGTGTGAAAT	19	[19]
S. enterica P*cuiD* (SctR)	TTGACCTTCCCGTTAGGGCAGGGTCTAAGCT	19	[20]
B. japonicum P*nolA* (NolA)	TTGAATCATACGTGACGTCAGGTTGTAGGCT	19	[21]
S.enterica P*copA* (CueR)	TTGACCTTAACCTTGCTGGAAGGTTTAACCT	19	[22]
S.enterica P*cueO* (CueR)	TTGACCTTCCCGTTAGGGCAGGGTCTAAGCT	19	[22]
S.enterica P*cueP* (CueR)	TTGACCTTTCCCTTAGGGGAACCCCTATAGT	19	[22]
S.enterica P*golB* (GolS)	TTGACCTTCCAACACTGGCAAGGTCCAGACT	19	[22]
S.enterica P*golTS* (GolS)	TTGACCTTCCCACAATGGCAAGCTTTAGGCT	19	[22]
S.enterica P*gesABC* (GolS)	TTGACCTTTCCTTCGTTGTAACGCCTAGCCT	19	[22]

Supplementary Table 3. Oligonucleotides with corresponding sequences (5' to 3') used for plasmid construction and promoter amplify.

Oligonucleotides	Sequences
pTolo-EX5 /*Bs*BmrR	TATTTTCAGGGCGCCATGGCCATGAAGGAATCGTATTACTCAATTGGGGTGG<br>TGGTGGTGGTGGTGCTCGAGTTATTCAGCGATCCGTATTTTCATTTCGAC
pET28a /*Bs*σ^A	TATTTTCAGGGCGCCATGGCCATGGCTGATAAACAAACCCACG<br>TGGTGGTGGTGGTGCTCGAGTTATTCAAGGAAATCTTTCAAACGTTTACTTCTG
pEASY /P-*bmr*	TTTGCAAATCCGTTGACTCTCCCC<br>AAATAAAAAGGCCTGCGATTACCAGCAGGCCTTAAGGTAATATTTTTCTTCTCCATATGAC
M13F:<br>M13R:	GTAAAACGACGGCCAGT<br>CAGGAAACAGCTATGAC

Supplementary Figures

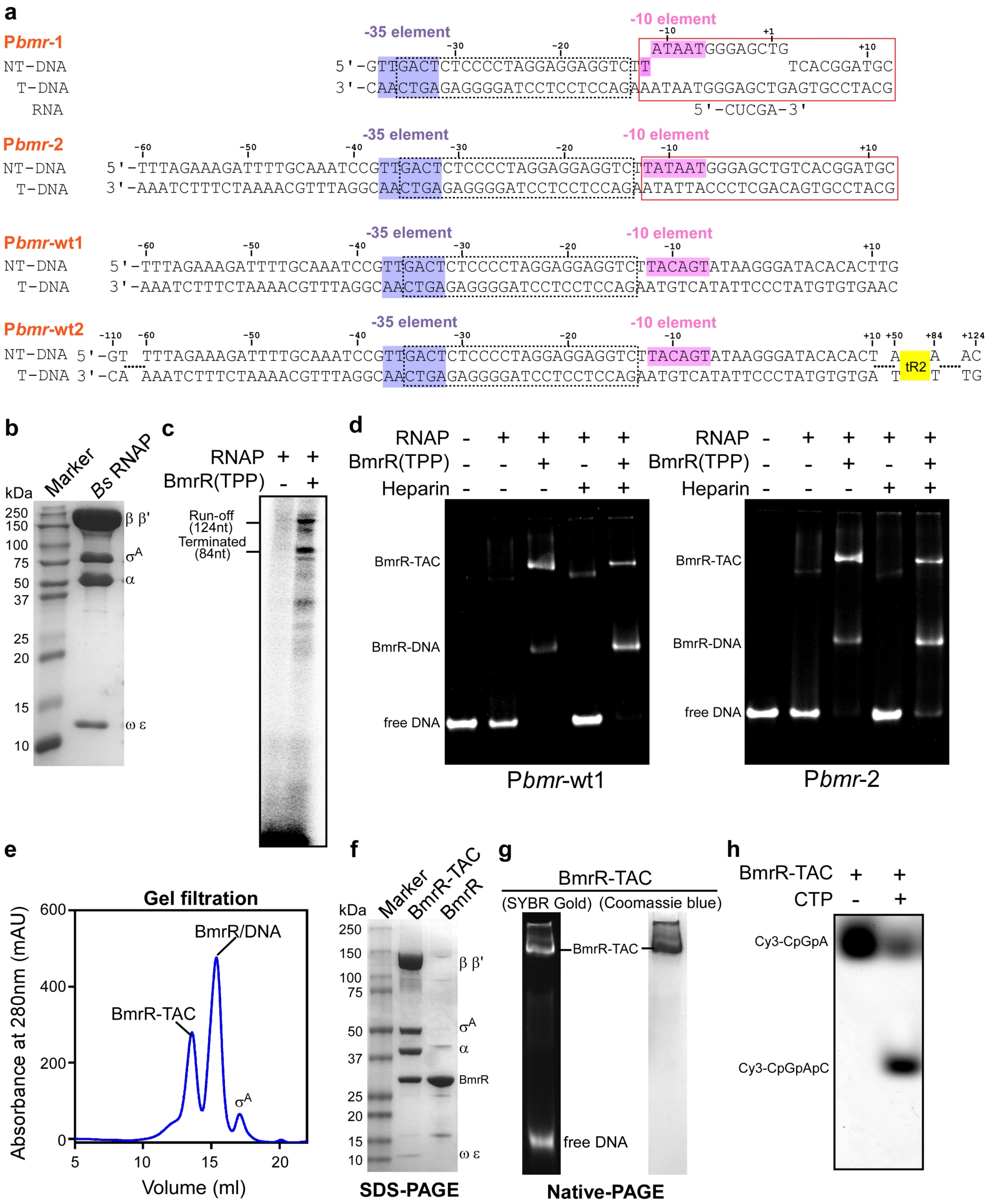

Supplementary Figure 1. The complex assembly of *B. subtilis* BmrR-TAC. (a) The promoters used in this study. P*bmr*-1 was used for cryo-EM structure determination of BmrR-TAC and for fluorescence-detected *in vitro* transcription assay in (h); P*bmr*-2 and P*bmr*-wt1 (a wild-type P*bmr*) were used for the gel-shift assay in (d); and P*bmr*-wt2 (a promoter containing wild-type P*bmr* sequence and a tR2 terminator) was used for radiochemical *in vitro* transcription assay in (c). The red box highlights regions with modified sequences. **(b)** The SDS-PAGE of endogenous *B. subtilis* RNAP holoenzyme. The experiment has been repeated >10 times. **(c)** The radiochemical *in vitro* transcription activity showing *B. subtilis* BmrR activates transcription from wild-type P*bmr* (Pbmr-wt2) promoter in the presence of 0.5 mM tetraphenylphosphonium (TPP). The experiment has been repeated independently twice. **(d)** BmrR increases the formation of RPo from the wild-type (P*bmr*-wt1) and derivative of P*bmr* (P*bmr*-2) promoters in the presence of 0.5 mM TPP. The experiment has been repeated independently three times. **(e)** The elution peak of *B. subtilis* BmrR-TAC from the Superose 6 size-exclusion column. **(f)** The SDS-PAGE of the *B. subtilis* BmrR-TAC, and BmrR. The experiment has been repeated twice. **(g)** The native-PAGE of the *B. subtilis* BmrR-TAC stained with Coomassie Brilliant Blue or SYBR Gold dye. The experiment has been repeated twice. **(h)** The fluorescence-detected *in vitro* transcription assay showing the reconstituted BmrR-TAC for cryo-EM study is able to extend 5' Cy3-labled CGA trinucleotide in the presence of CTP. The identity of Cy3-CGA and Cy3-CGAC was further confirmed by LC-MS/MS. The slower migration of 3-nt RNA oligomer compared with 4-nt RNA oligomer has also been observed in primer-dependent transcription initiation using a 5'-OH RNA primer [23]. The experiment has been repeated 3 times.

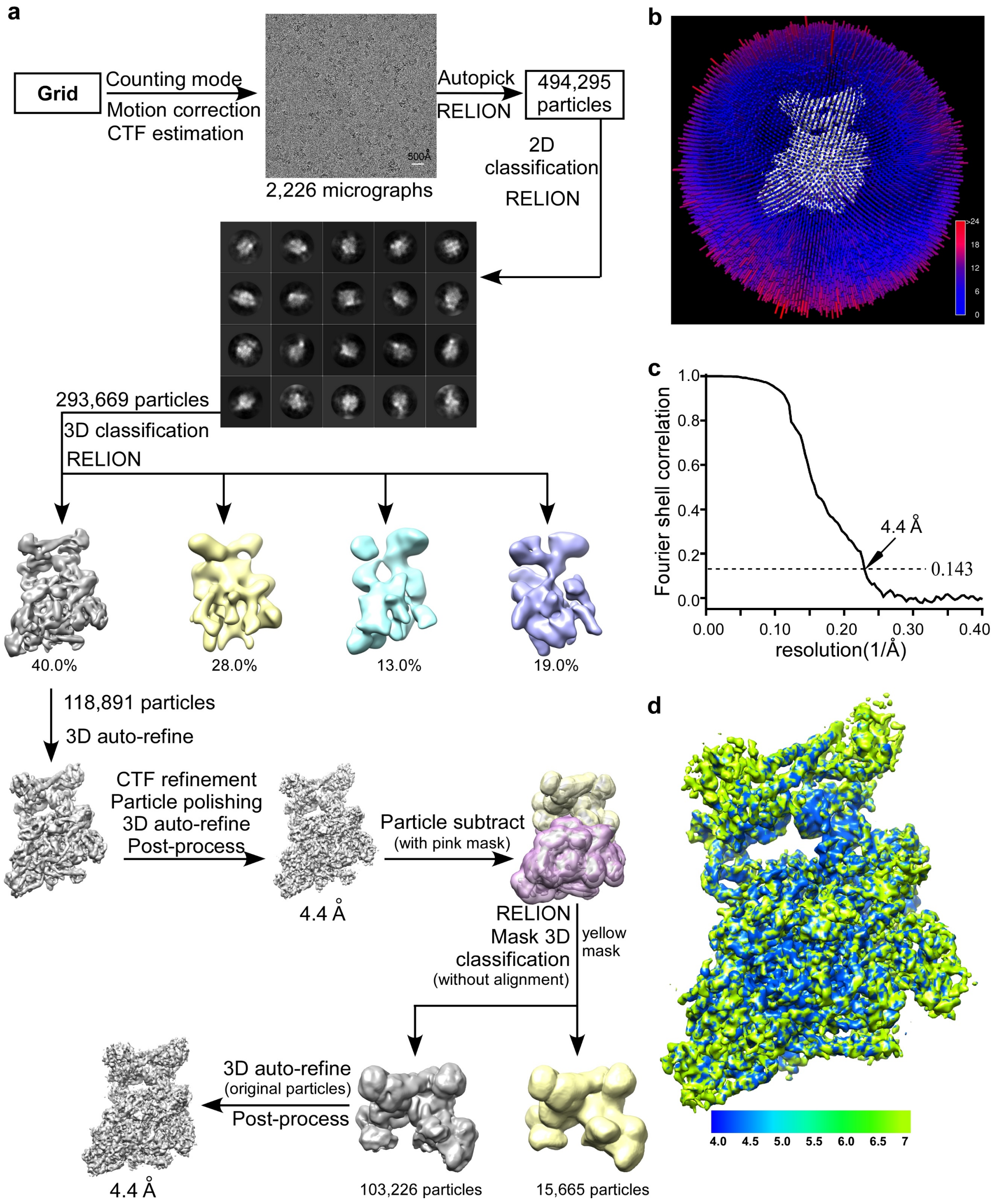

Supplementary Figure 2. The processing pipeline for single-particle reconstitution of cryo-EM map of *B. subtilis* BmrR-TAC. (a) The flowchart of data processing. **(b)** The angular distribution of *B. subtilis* BmrR-TAC single-particle projections colored by number of particles of each projection. **(c)** The gold-standard FSC of *B. subtilis* BmrR-TAC model reconstruction. The 0.143 FSC cutoff indicates a nominal resolution of 4.40 Å. **(d)** The cryo-EM map of *B. subtilis* BmrR-TAC colored by local resolution (calculated by Resmap).

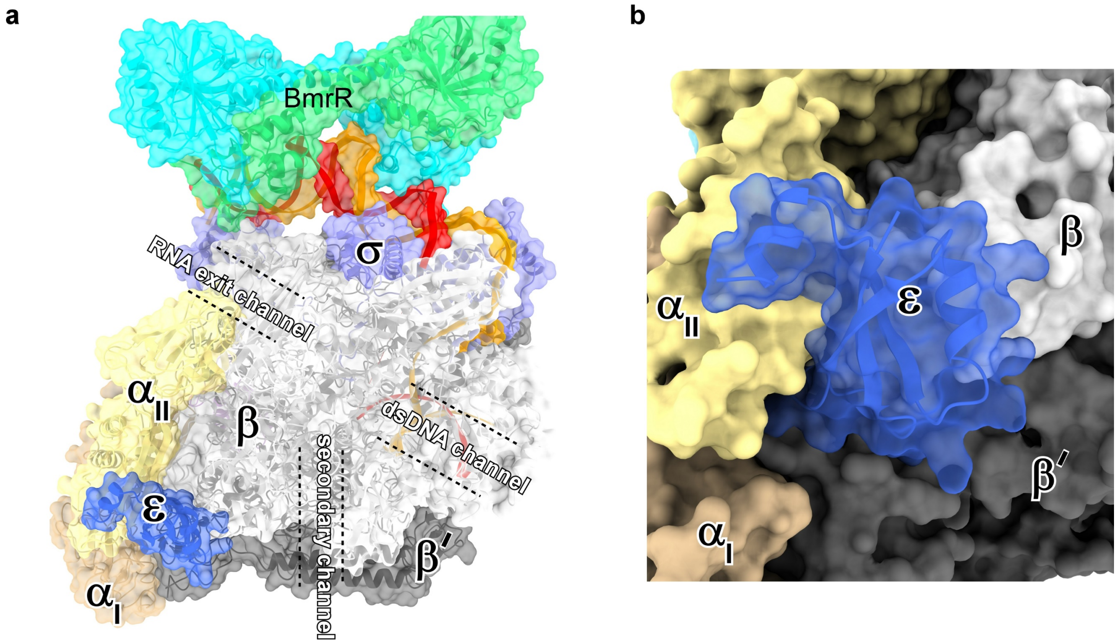

Supplementary Figure 3. The interaction between RNAP-ε subunit and the rest of RNAP core enzyme. (a) The overall structure displays the location of RNAP-ε subunit on RNAP. **(b)** The RNAP-ε subunit makes extensive interaction with RNAP-α, β and β' subunits.

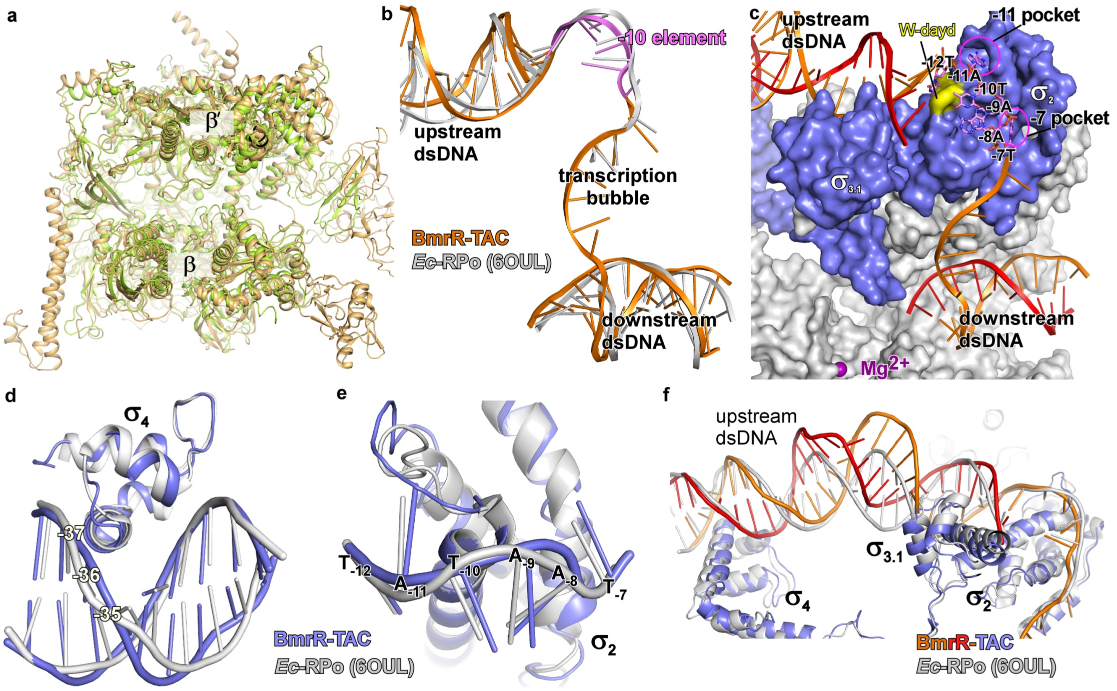

Supplementary Figure 4. The BmrR-TAC has characteristics of RPo. (a) The BmrR-TAC structure exhibits the same closed conformation of RNAP-clamp domain as that in *E. coli* RPo complex (PDB: 6CA0; [24]). **(b)** The BmrR-TAC structure exhibits the same DNA conformation of the transcription bubble and the downstream dsDNA (PDB: 6OUL; [25]). **(c)** The *B. subtilis* RNAP-holoenzyme accommodates the transcription bubble and downstream dsDNA in a similar manner as other bacterial RNAP. The W-dyad separates the upstream junction and the -11 and -7 pockets accommodate flipped bases in the structure. **(d)** The σ_4/-35 interaction in the BmrR-TAC is similar to *E. coli* σ_4/-35 DNA interaction (PDB: 6OUL; [25]). **(e)** The σ_2/-10 interaction in the BmrR-TAC is similar to *E. coli* σ_2/-10 DNA interaction (PDB: 6OUL; [25]). **(f)** The locations of σ_2 and σ_4/ on RNAP core enzyme in BmrR-TAC are similar compared with the *E. coli* transcription open complex (PDB: 6OUL; [25]) with r.m.s.d. values of 1.95 Å for all σ_2/σ_4 Cα atoms of the two structures.

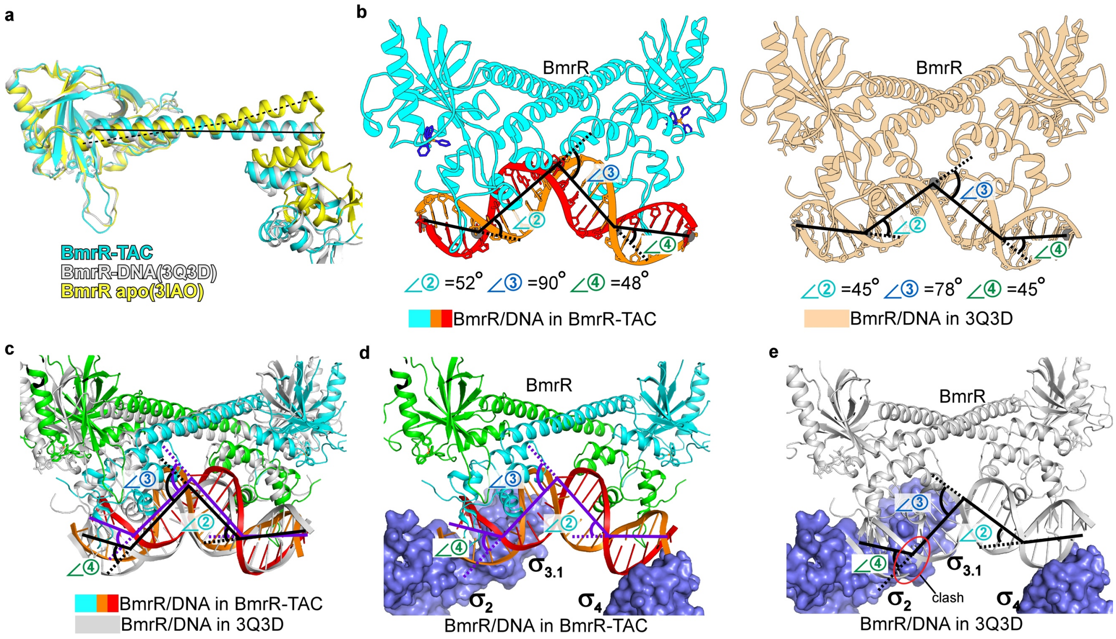

Supplementary Figure 5. The comparison of the BmrR-TAC structure with previous reported crystal structures. (a) The central helix adopts the straight conformation for activation in BmrR-TAC (cyan) as in the structure of BmrR-DNA (PDB: 1EXI; gray) compared with the inactive bent conformation in the BmrR apo structure (PDB: 3IAO). **(b)** Kinks 2, 3, and 4 pre-exist in the binary structure of BmrR-DNA (PDB: 3Q3D) and are further enlarged upon RNAP engagement. **(c)** The BmrR-DNA (PDB: 3Q3D) superimposed based on the -35 element with BmrR-TAC show slightly different paths of the downstream dsDNA due to different kink angles resulting in a steric clash of the promoter DNA with σ as shown in (e). **(d)** The σ_4 and $\sigma_{3.1}$ together induce slightly enlargement of kinks 2, 3, and 4 in BmrR-TAC. **(e)** Structure modeling of BmrR-DNA (PDB: 3Q3D) onto RNAP holoenzyme, based on -35 element superimposition of BmrR-DNA and BmrR-TAC, implicates a steric clash between the promoter DNA and σ, suggesting the necessity of slight enlargement of the three kinks during promoter engagement of RNAP holoenzyme.

Supplementary References

1. Barrineau, P. et al. The DNA sequence of the mercury resistance operon of the IncFII plasmid NR1. *J Mol Appl Genet* **2**, 601-19 (1984).
2. Helmann, J.D., Wang, Y., Mahler, I. & Walsh, C.T. Homologous metalloregulatory proteins from both gram-positive and gram-negative bacteria control transcription of mercury resistance operons. *J Bacteriol* **171**, 222-9 (1989).
3. Laddaga, R.A., Chu, L., Misra, T.K. & Silver, S. Nucleotide sequence and expression of the mercurial-resistance operon from Staphylococcus aureus plasmid pI258. *Proc Natl Acad Sci U S A* **84**, 5106-10 (1987).
4. Brocklehurst, K.R. et al. ZntR is a Zn(II)-responsive MerR-like transcriptional regulator of zntA in Escherichia coli. *Mol Microbiol* **31**, 893-902 (1999).
5. Outten, F.W., Outten, C.E., Hale, J. & O'Halloran, T.V. Transcriptional activation of an Escherichia coli copper efflux regulon by the chromosomal MerR homologue, cueR. *J Biol Chem* **275**, 31024-9 (2000).
6. Rutherford, J.C., Cavet, J.S. & Robinson, N.J. Cobalt-dependent transcriptional switching by a dual-effector MerR-like protein regulates a cobalt-exporting variant CPx-type ATPase. *J Biol Chem* **274**, 25827-32 (1999).
7. Borremans, B., Hobman, J.L., Provoost, A., Brown, N.L. & van Der Lelie, D. Cloning and functional analysis of the pbr lead resistance determinant of Ralstonia metallidurans CH34. *J Bacteriol* **183**, 5651-8 (2001).
8. Lee, S.W., Glickmann, E. & Cooksey, D.A. Chromosomal locus for cadmium resistance in Pseudomonas putida consisting of a cadmium-transporting ATPase and a MerR family response regulator. *Appl Environ Microbiol* **67**, 1437-44 (2001).
9. Hidalgo, E. & Demple, B. An iron-sulfur center essential for transcriptional activation by the redox-sensing SoxR protein. *Embo j* **13**, 138-46 (1994).
10. Holmes, D.J., Caso, J.L. & Thompson, C.J. Autogenous transcriptional activation of a thiostrepton-induced gene in Streptomyces lividans. *Embo j* **12**, 3183-91 (1993).
11. Ahmed, M., Borsch, C.M., Taylor, S.S., Vazquez-Laslop, N. & Neyfakh, A.A. A protein that activates expression of a multidrug efflux transporter upon binding the transporter substrates. *J Biol Chem* **269**, 28506-13 (1994).
12. Ahmed, M. et al. Two highly similar multidrug transporters of Bacillus subtilis whose expression is differentially regulated. *J Bacteriol* **177**, 3904-10 (1995).
13. Newberry, K.J. & Brennan, R.G. The structural mechanism for transcription activation by MerR family member multidrug transporter activation, N terminus. *J Biol Chem* **279**, 20356-62 (2004).
14. Chambers, J.R., Liao, J., Schurr, M.J. & Sauer, K. BrlR from Pseudomonas aeruginosa is a c-di-GMP-responsive transcription factor. *Mol Microbiol* **92**, 471-87 (2014).
15. Kidd, S.P., Potter, A.J., Apicella, M.A., Jennings, M.P. & McEwan, A.G. NmlR of Neisseria gonorrhoeae: a novel redox responsive transcription factor from the MerR family. *Mol Microbiol* **57**, 1676-89 (2005).
16. Peuser, V., Glaeser, J. & Klug, G. The RSP_2889 gene product of Rhodobacter sphaeroides is a CueR homologue controlling copper-responsive genes. *Microbiology* **157**, 3306-13 (2011).
17. Kidd, S.P. et al. A novel nickel responsive MerR-like regulator, NimR, from Haemophilus influenzae. *Metallomics* **3**, 1009-18 (2011).
18. Julian, D.J., Kershaw, C.J., Brown, N.L. & Hobman, J.L. Transcriptional activation of MerR family promoters in Cupriavidus metallidurans CH34. *Antonie Van Leeuwenhoek* **96**, 149-59 (2009).
19. Kidd, S.P. & Brown, N.L. ZccR—a MerR-like regulator from Bordetella pertussis which responds to zinc, cadmium, and cobalt. *Biochemical and Biophysical Research Communications* **302**, 697-702 (2003).

20. Kim, J.S. et al. The sctR of Salmonella enterica serova Typhimurium encoding a homologue of MerR protein is involved in the copper-responsive regulation of cuiD. *FEMS Microbiol Lett* **210**, 99-103 (2002).
21. Loh, J. & Stacey, G. Nodulation gene regulation in Bradyrhizobium japonicum: a unique integration of global regulatory circuits. *Appl Environ Microbiol* **69**, 10-7 (2003).
22. Humbert, M.V., Rasia, R.M., Checa, S.K. & Soncini, F.C. Protein signatures that promote operator selectivity among paralog MerR monovalent metal ion regulators. *J Biol Chem* **288**, 20510-9 (2013).
23. Zhang, Y. et al. GE23077 binds to the RNA polymerase 'i' and 'i+1' sites and prevents the binding of initiating nucleotides. *Elife* **3**, e02450 (2014).
24. Narayanan, A. et al. Cryo-EM structure of Escherichia coli sigma(70) RNA polymerase and promoter DNA complex revealed a role of sigma non-conserved region during the open complex formation. *J Biol Chem* **293**, 7367-7375 (2018).
25. Chen, J. et al. E. coli TraR allosterically regulates transcription initiation by altering RNA polymerase conformation. *Elife* **8**(2019).
